# *Culex quinquefasciatus* Late Trypsin Biosynthesis Is Translationally Regulated by Trypsin Modulating Oostatic Factor

**DOI:** 10.3389/fphys.2021.764061

**Published:** 2021-11-17

**Authors:** Dov Borovsky, Peter Verhaert, Pierre Rougé, Charles A. Powell, Arnold De Loof

**Affiliations:** ^1^Department of Biochemistry and Molecular Genetics, University of Colorado Anschutz Medical Campus, Aurora, CO, United States; ^2^ProteoFormiX BV, Beerse, Belgium; ^3^UMR 152 Pharma-Dev, Institut de Recherche et Développement, Université Toulouse 3, Faculté des Sciences Pharmaceutiques, Toulouse, France; ^4^UF-IFAS Indian River Research and Education Center, Fort Pierce, FL, United States; ^5^Zoological Institute, KU Leuven, Leuven, Belgium

**Keywords:** *Culex quinquefasciatus*, late trypsin biosynthesis, cloning and sequencing, three-dimensional modeling, blood digestion

## Abstract

Trypsin is a serine protease that is synthesized by the gut epithelial cells of female mosquitoes; it is the enzyme that digests the blood meal. To study its molecular regulation, *Culex quinquefasciatus* late trypsin was purified by diethylaminoethyl (DEAE), affinity, and C_18_ reverse-phase high performance liquid chromatography (HPLC) steps, and the N-terminal amino acid sequence was determined for molecular cloning. Five overlapping segments of the late trypsin cDNA were amplified by PCR, cloned, and the full sequence (855 bp) was characterized. Three-dimensional models of the pro-trypsin and activated trypsin were built and compared with other trypsin models. Trypsin modulating oostatic factor (TMOF) concentrations in the hemolymph were determined by ELISA and compared with trypsin activity in the gut after the blood meal. The results showed that there was an increase in TMOF concentrations circulating in the hemolymph which has correlated to the reduction of trypsin activity in the mosquito gut. Northern blot analysis of the trypsin transcripts after the blood meal indicated that trypsin activity also followed the increase and decrease of the trypsin transcript. Injections of different amounts of TMOF (0.025 to 50 μg) decreased the amounts of trypsin in the gut. However, Northern blot analysis showed that TMOF injections did not cause a decrease in trypsin transcript abundance, indicating that TMOF probably affected trypsin translation.

## Introduction

Anautogenous female mosquitoes take a blood meal to develop their eggs in the ovaries and are important vectors of human infectious diseases such as malaria, dengue, and yellow fever. *Culex quinquefasciatus* found in subtropical regions feeds on blood from humans, dogs, birds, and livestock, thus being adapted to transmit arboviruses between humans and animals such as West Nile, St. Louis encephalitis, and Venezuelan equine encephalitis viruses (Farajollahi et al., 2011; Simpson et al., 2012). To digest the blood meal to free amino acids female mosquitoes, synthesize trypsin, and chymotrypsin-like enzymes (Borovsky, 2003). The blood digestion in female mosquitoes is a two-phase process regulated at the midgut epithelial cells. Following the blood meal, trypsin biosynthesis is stimulated by soluble proteins that activate a pre-existing trypsin transcript of the early trypsin in both *Aedes aegypti* and *C. quinquefasciatus* (Felix et al., 1991; Borovsky et al., 2018). Female *C. quinquefasciatus* that were fed on sugar before entering diapause were reported to also synthesize trypsin-like enzymes in their midgut. These enzymes were identified by sodium dodecyl sulfate polyacrylamide gel electrophoresis (SDS-PAGE) followed by tandem mass spectrometry (MS/MS) analyses or by molecular cloning and characterization. These studies suggested that the early trypsin might play a role in the activation of the late trypsin after the blood meal. However, their role in digesting the blood meal or whether they are continued to be synthesized or downregulated after the blood remains unknown (Robitch and Denlinger, 2005; Borges-Veloso et al., 2015).

Earlier reports (Barillas-Mury et al., 1995; Noriega and Wells, 1999) suggested that in *A. aegypti*, the early trypsin and juvenile hormone (JH) were required for the transcription and subsequent translation of the late trypsin. These claims were shown to be incorrect (Borovsky, 2003; Lu et al., 2006) indicating that an unknown mechanism, which is yet to be discovered, possibly controls the mosquito early and late trypsin. The role of JH in the biosynthesis of the early trypsin in *C. quinquefasciatus* was reported several years ago (Borovsky et al., 2018). These authors showed that JH controlled the splicing of the early trypsin transcript that was covalently bound to RNA ribonucleoprotein (RNP) by a phosphoamide bond at the 5′-end of the transcript, and that the early trypsin biosynthesis depended on sugar and blood-feeding, whereas the late trypsin biosynthesis did not depend on sugar feeding or JH. Downregulating *C. quinquefasciatus* early trypsin transcript does not affect the late trypsin. Only blood digestion controls the late trypsin translation as was shown in *A. aegypti* (Borovsky, 2003; Lu et al., 2006).

Trypsin modulating oostatic factor (TMOF), a decapeptide (YDPAPPPPPP) synthesized by female *A. aegypti* ovary, controls the biosynthesis of trypsin-like enzymes in the midgut epithelial cells of several species of mosquito including *A. aegypti*, *C. quinquefasciatus*, *C*ule*x nigripalpus*, and *Anopheles albimanus* (Borovsky, 1988). Using immunochemistry, we showed that *Aea*TMOF like peptide was circulating in the hemolymph of female *C. quinquefasciatus* after the blood meal. We purified the *C. quinquefasciatus* late trypsin, cloned its cDNA, and molecularly characterized the late trypsin gene. We demonstrate that *Aea*TMOF affected the translation of the late trypsin transcript in the gut epithelial cells as it has been shown for *Neobellieria* in which *Neo*TMOF (10^–9^ M), a hexapeptide (NPTNLH) different than *Aea*TMOF, inhibited the translation of the late trypsin transcript (Borovsky et al., 1996). Similarly, in *Heliothis virescens*, *Aea*TMOF engineered on the coat protein of Tobacco Mosaic Virus stopped the translation of the trypsin transcript in the gut epithelial cells (Borovsky et al., 2006). To clone and sequence the *C. quinquefasciatus* late trypsin, we combined biochemical and molecular techniques to first purify the enzyme by diethylaminoethyl (DEAE) and affinity chromatography followed by high performance liquid chromatography (HPLC). The purified enzyme N-terminal was sequenced allowing us to design primers for cloning and molecular sequencing the late trypsin as was successfully reported for the trypsin of *Neobellieria bullata* (Borovsky et al., 1996). This report shows for the first time that in female *C. Quinquefasciatus*, TMOF affects trypsin activity by inhibiting the translation of the late trypsin transcript.

## Materials and Methods

### Experimental Insects

Larval *C. quinquefasciatus* were reared at 27°C in the laboratory on a diet of lactalbumin and yeast extract (1:1), under 16:8 h light:dark cycle. Adults were fed on 10% sucrose and chicken blood and used 1–3 days after emergence.

### Injections and Surgical Manipulations

Female *C. quinquefasciatus* were blood-fed lightly anesthetized by ether and immediately injected (0.25 μl) with different concentrations of TMOF or water (control) with a finely drawn glass capillary between the second and third segments of the female abdomen. After injections, females were kept in a cage lined with tissue paper in a bio room at 37°C and 80% humidity, then analyzed 30 h later.

### Reagents

N_α_-benzoyl-DL-arginine-4-nitroanilide (BApNA) was purchased from Sigma (St. Louis, MO, United States). TMOF was synthesized and purified by HPLC, and the peptide was analyzed by MS as described previously (Borovsky et al., 1990). Radioactively labeled [1,3-^3^H]DFP (5 μCi) specific activity 35 Ci mmol for labeling trypsin (Borovsky and Schlein, 1988) was purchased from Amersham (Arlington Heights, IL, United States).

### Enzyme Assay and Biosynthesis

Trypsin activity was measured with BApNA. Enzyme aliquots were incubated for 30 min at 30°C, and absorbance was read at 410 nm as was described previously (Borovsky and Schlein, 1988). For pH activity profile the following buffers were used: 0.2 M citric/phosphate (pH 3.2–7.2); 0.2 M Tris–HCl (pH 7.2–8.9); 0.2 glycine/NaOH (pH 8.9–10.9). The amount of trypsin synthesized per female gut after the blood meal was determined by incubating [1,3-^3^H]DFP with trypsin and the [1,3-^3^H]DIP-trypsin derivatives were measured (Borovsky and Schlein, 1988).

### ELISA

**FIGURE 1:**
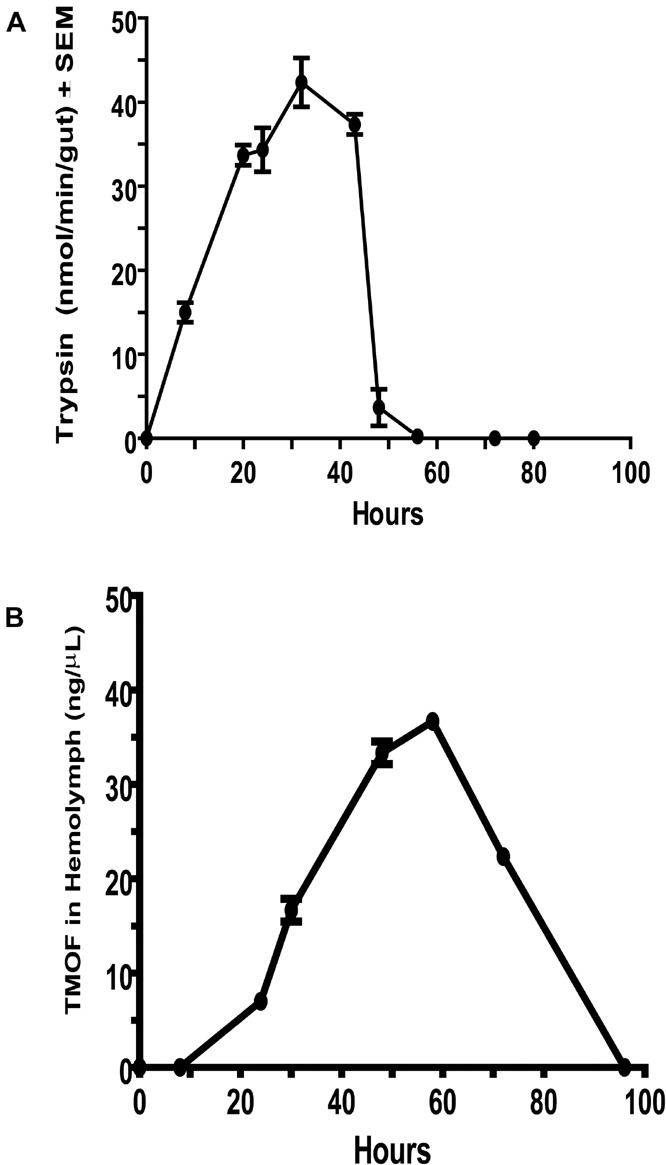
Comparison between *Culex quinquefasciatus* late trypsin activity in the gut and trypsin modulating oostatic factor (TMOF) concentrations in the hemolymph. **(A)** Trypsin activity at different times after the blood meal was followed using BApNA in female guts. Activity is expressed as nmol/min/gut, which are then expressed as means of 3 determinations ±SEM. **(B)** The concentration of *Aea*TMOF in the hemolymph of female mosquitoes was followed at intervals after the blood meal using ELISA. Each concentration represents the mean of 3 determination ±SEM.

Female *C. quinquefasciatus* hemolymph samples were collected (3 groups of 100 females each) by centrifugation at different times after the blood meal after removing the female legs. The legless females were transferred into Eppendorf tubes fitted with a filter paper separating the legless females from the bottom of the Eppendorf tube. The legless females were centrifuged at 5,000 rpm in an Eppendorf microcentrifuge for 10 min at 4°C. After centrifugation, the hemolymph was collected from the bottom of the tubes (about 0.25 μl/female) and assayed for TMOF using ELISA (Borovsky et al., 1992). Each determination was repeated three times and the results are expressed as the average of 3 determinations ±SEM (ng/μl ±SEM) (Figure 1).

### Anion Exchange Column Chromatography

**FIGURE 2:**
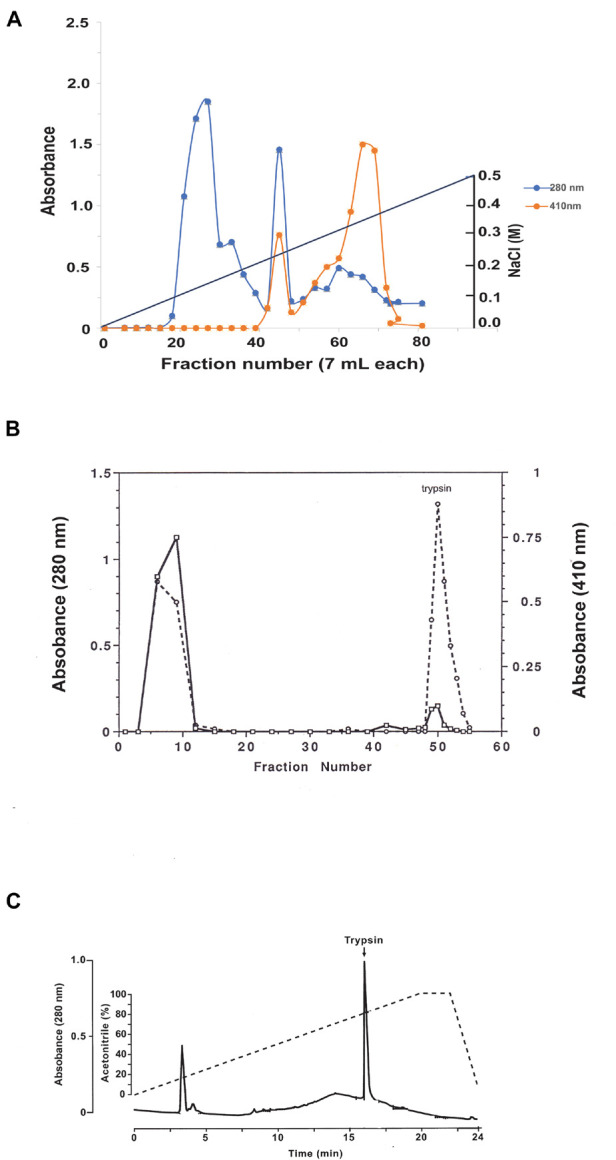
Purification of *C. quinquefasciatus* late trypsin using column chromatography. **(A)** Purification using DEAE chromatography. Protein absorbance at 280 nm is shown in the blue line. Trypsin activity was followed using BApNA monitored at 410 nm (orange line). NaCl elution gradient is shown as a black line. **(B)** Affinity chromatography of *C. quinquefasciatus* late trypsin on Soybean trypsin inhibitor Sepharose-4B column. Late trypsin was eluted with 20 mM Glycine-HCl pH 3.0 buffer. Protein was followed by absorbance at 280 nm (solid black line) and trypsin activity was followed using BApNA at 410 nm (broken line). **(C)** Reverse phase C_18_ HPLC. Protein was eluted using a linear gradient of acetonitrile in the presence of.1% TFA (dashed line). The column was calibrated with [1,3-^3^H]DIP trypsin (arrow). Collected fractions were monitored for protein at 280 nm (solid line) the late trypsin was found to elute at 16.04 min.

Female *C. quinquefasciatus* were fed a blood meal and 30 h later guts were surgically dissected from 300 females under a dissecting microscope, homogenized in an Eppendorf tube (2 ml) at 4°C with 50 mM Tris–HCl pH 7.9 buffer (1.5 ml), centrifuged at 14,000 rpm in a micro-centrifuge for 10 min at 4°C, and the supernatant was removed. A DEAE biogel (Bio-Rad, Hercules, CA, United States) column (1.5 cm × 91 cm) was equilibrated with 50 mM Tris–HCl pH 7.9 at 4°C, and 1.5 ml of the supernatant was adsorbed onto the DEAE biogel and eluted with a linear gradient of NaCl (500 ml, 0–0.5 M) at a speed of 3.5 ml/h. Fractions (7 ml) were collected and aliquots (200 μl) were assayed for trypsin activity using BApNA (Borovsky and Schlein, 1988; Figure 2A).

### Affinity Chromatography

The major peak with trypsin activity (70 ml) that was eluted at 0.35 M (Figure 2A) was collected, dialyzed against 50 mM Tris–HCl pH 7.9 (2,000 ml) at 4°C overnight, and adsorbed to 5 ml of soybean trypsin inhibitor sepharose 4B packed into a column (0.5 cm × 10 cm) that was previously calibrated with 50 mM Tris–HCl pH 7.9 buffer. The column was then washed at 4°C with the same equilibrating buffer (12 ml) and then eluted with 35 ml of 20 mM Glycine-HCl pH 3 buffer into tubes containing 1 M Tris–HCl pH 7.9 (1 ml). Fractions (1 ml) were collected and aliquots (100 μl) were assayed for trypsin activity at 410 nm using BApNA (Borovsky and Schlein, 1988), and eluted proteins were followed by their 280 nm absorbance (Figure 2B). Fractions 49–54 (12 ml) were combined, and the volume was concentrated to 1.5 ml using an Amicon Diaflo concentrating cell at 4°C with a membrane PM 10 cut-off M_*r*_ = 10 kDa (Sigma, St. Louis MO, United States).

### Reverse Phase C_18_ High Performance Liquid Chromatography

Aliquots at 200 μl from the affinity column concentrate (1.5 ml, as described above) were chromatographed on HPLC system using microsorb 5 μm C_18_ reverse-phase column (4.6 mm i.d. by 25 cm) and a 5 mm guard cartridge. The column was eluted using a gradient of acetonitrile (0–100%) in 0.1% trifluoroacetic acid (TFA) (Borovsky and Mahmood, 1995). The column was calibrated with [1,3-^3^H]DIP labeled trypsin synthesized after the affinity chromatography. Briefly, aliquots (100 μl) from the concentrated trypsin peak after affinity chromatography (see above) were incubated with [1,3-^3^H]DFP (Borovsky and Schlein, 1988) and the [1,3-^3^H]DIP labeled trypsin derivative were purified by SepPak C_18_ reverse-phase mini-column (Waters, Milford, MA, United States) by eluting the unreacted [1,3-^3^H]DFP in 10% acetonitrile 0.1% TFA (4 ml) and then the [1,3-^3^H]DIP labeled trypsin in 100% acetonitrile 0.1% TFA (4 ml). The eluted [1,3-^3^H]DFP-trypsin radioactivity was determined using a liquid scintillation counter (Borovsky et al., 2021), dried under a fine stream on N_2_, rehydrated in water 0.1% TFA (100 μl), and used to calibrate the HPLC column (Figure 2C). The HPLC chromatography was repeated four times and the eluted trypsin peaks combined and the N-terminal sequence of the late *C. quinquefasciatus* trypsin was determined.

### N-Terminal Amino Acids Sequencing

Total protein content was determined according to Bradford (1976) and bovine serum albumin as a standard. Aliquots from the pooled four samples (200 μg each) after HPLC purification were assayed to determine the N-terminal sequence of *C. quinquefasciatus* later trypsin (6.3 pmol) by Edman degradation on a pulsed liquid protein sequencer (Beckman LF3200). Phenylthiohydantoin (PTH)-amino acids were separated in an acetonitrile gradient on a micro-C_18_ RP-HPLC column kept at constant temperature in a column oven (52°C). The analysis ran 30 Edman degradation cycles. The first 19 cycles yielded the unambiguous N-terminal sequence IVGGFEIDILEVPYQISL.

### RNA Extraction

Four groups of female *C. quinquefasciatus* (500 per group) were fed sugar for 2 days and fed blood on a chicken. Then, at intervals (4, 30, 50, and 60 h) their guts were surgically removed using a dissecting microscope into a drop of saline. The gut was opened, the blood bolus removed, the gut epithelial cells were washed in phosphate-buffered saline (PBS) pH 7.2 and homogenized in 1 ml TRIzol (ThermoFisher, Carlsbad, CA, United States). Total RNA was prepared from the TRIzol homogenate for each group following the instructions of the manufacturer. The RNA was stored in RNase-free TE buffer, pH 8 at −20°C until used (Borovsky et al., 1996, 2021; Yan et al., 1999).

### Northern Blot Analysis

Ambion Northern Max kit was used for the Northern blot analyses (Ambion, Austin, TX, United States). RNA samples from isolated gut epithelial cells (15 μg per lane) that were extracted at different times after the blood meal and from sugar-fed females, as well as from blood-fed females that were injected with different amounts of TMOF (0.025–50 μg) or water control, were separated on denaturing 1% formaldehyde agarose gel at 100 V for 1.5 h and transferred to Hydrobond-N^+^ nylon membrane (Borovsky et al., 1996, 2018). The membranes were hybridized with [^32^P]-labeled DNA probe (294 nt) (Supplementary Material) that was prepared by PCR using primers DB 102 and DB 99 (Table 1), a random prime kit was used to label the DNA with [^32^P]dCTP following the instructions of the manufacturer (Agilent, Santa Clara, CA, United States). Membranes were exposed to X-ray film for 48 h at −80°C and then developed (Borovsky et al., 1996; Yan et al., 1999). Radioactively labeled probes were stripped from the membrane in a boiling solution of 0.1% SDS and the membrane was scanned with a Geiger counter to confirm that the probes were completely removed. *C. quinquefasciatus* actin transcript probe (Borovsky et al., 2018) was used to show the transfers to the membranes in control and experimental lanes. The Northern blot analyses were repeated twice showing similar results. Since the transfer of *actin* transcript in each lane was not equal, the Northern blots were normalized by scanning the blot using Sapphire Biomolecular Imager (Azure Biosystems, Dublin, CA, United States) using three wavelengths (488, 520, and 658 nm) and the opacity of the scanned *trypsin* transcript bands were divided by the opacity of the scanned *actin* transcript bands that were used as reference genes. The ratios were then plotted against different amounts of injected TMOF (0.025–50 μg) and water control and were used to study the abundance of the *trypsin* transcripts in injected female *C. quinquefasciatus* as compared with water-injected control.

**TABLE 1:** Primers used in sequencing and Northern blot analysis of *Culex quinquefasciatus* late trypsin transcript.

Primers	Primers sequence (5′-3′)	Position (5′-3′)	Amplicon (nt)	*tm* (°C)
*5*′ *RACE*				
dT_17_ adapter (forward)	GACTCGAG TCGACATCGA(T)_17_			74
DB 80 (reverse)	CAAAACCCAGT TTTCACC	(−30) to 225	255	49
DB 73 (reverse)	GAGTCCAACGT CGTTATCATC	1–264	264	54
*3*′ *RACE*				
DB 75 (forward)	TACGGAGGCTACCA GATTACCGAT			59
dT_17_ adapter (reverse)	GACTCGAGTC GACATCGA(T)_17_	576–855	279	74
Adapter (reverse)	GACTCGAGTC GACATCGA			66
DB 46 (forward)	ATHGTIGGIGG ITTYGAR			45
DB 66 (reverse)	ICCICCIGARTCI CCYTGRCA	125–717	592	57
DB 95 (reverse)	GATACCACTGTG CTCCTTGAC	125–789	664	55
*Northern blot*				
DB 102 (forward)	TGATCATTCA GTTCAGAG			46
DB 99 (reverse)	GAGTCCAACGT CGTTATC	(−30) to 264	294	50

*Degenerate nucleotides coding: R = A, G; H = T, C, A; Y = T, C; I = inosine. Degenerate primers nucleotides were synthesized as mixtures and were used for RT−PCR to amplify the Culex quinquefasciatus late trypsin messenger RNA found in adult female gut epithelial cells 30 h after the blood meal.*

### PCR

Primers were synthesized by Sigma-Aldrich, and their positions and *t*_*m*_ on the *C. quinquefasciatus* late trypsin transcript are listed in Table 1. The forward and reverse sequences of primers DB 46 and DB 66 were derived from the N-terminal sequencing of the late trypsin and the active site sequences of *A. aegypti*, *Anopheles gambiae*, *Manduca sexta*, *Drosophila melanogaster*, and *C. quinquefasciatus* early trypsin (Davis et al., 1985; Kalhok et al., 1993; Muller et al., 1993; Peterson et al., 1994; Borovsky et al., 2018). PCR conditions for RT-PCR and PCR to amplify the late *C. quinquefasciatus* trypsin were similar as was reported for *N. bullata*, *Diaprepes abbreviatus* and *C. quinquefasciatus* early trypsin (Borovsky et al., 1996, 2018; Yan et al., 1999). For rapid amplification of the 3′ and 5′ cDNA ends (RACE), the forward and reverse primer sets are described in Table 1, and the PCR conditions were the same as those that were described before (Borovsky et al., 1996; Yan et al., 1999).

### Cloning and Sequencing

The cDNA segments produced by PCR amplification of gut epithelial cells RNA were subcloned into pCR2.1 vector using TA cloning kit (Invitrogen, Waltham, MA, United States). The plasmid DNA was purified using QIAprep Spin Miniprep Kit, digested to release cloned fragments, analyzed by electrophoresis, and sequenced (Borovsky et al., 1996; Yan et al., 1999). Sequences were analyzed with DNAstar v.12 software (DNAstar, Madison, WI, United States).

### Three-Dimensional Modeling

Homology modeling of the *C. quinquefasciatus* late pro-trypsin was performed with the YASARA Structure program (Krieger et al., 2002). Different templates of pro-trypsin were used to build the pro-trypsin model using the atomic coordinates of trypsin-1 from the Atlantic salmon (*Salmo salar*) (PDB code 1HJ8 and 1UTJ) (Leiros et al., 2001, 2004), anionic trypsin (PDB code 2ZPQ), cationic trypsin isoform 2 (PDB code 2ZPR), and anionic trypsin isoform 3 (PDB code 2ZPS) from the chum salmon (*Oncorhynchus keta*) (Toyota et al., 2009). The model that was built using 2ZPR, contained 233 amino acid residues (7–240), exhibited a better *Z*-score, and was saved as the final model for the late *C. quinquefasciatus* pro-trypsin. PROCHECK (Laskowski et al., 1993), ANOLEA (Melo and Feytmans, 1998), and the calculated QMEAN scores (Benkert et al., 2011; Grosdidier et al., 2011), were used to assess the geometric and thermodynamic qualities of the model. Only three residues (R16, Y37, and H74) over 234, occurred in the non-allowed regions in the Ramachandran plot. Using ANOLEA to evaluate the model, 33 residues (out of a total of 234) of the model exhibited energy over the threshold value. Stretches of residues exhibiting the higher values corresponded to the pro-peptide from the pro-trypsin and some extended loops that protrude from the protein core, surrounding the central catalytic groove. The calculated QMEAN score for the model was −1.33. The activated trypsin, a polypeptide chain of 227 amino acids devoid of the activation peptide, was similarly modeled from the atomic coordinates of the same template proteins. A model that was built from the 2ZPQ template (anionic trypsin from the chum salmon), containing 224 amino acid residues (1–224) and was saved as the final model for the cleaved trypsin of *C. quinquefasciatus*. A single residue (H58) of the model built for the activated trypsin occurred in the non-allowed region of the Ramachandran plot. Using ANOLEA to evaluate the model, only six residues (out of 224) of the model exhibited energy over the threshold value and the QMEAN score for the model was 0.1.

### Statistical Analysis

Data were analyzed using Graph Pad Prism v.5 using a two-tailed unpaired *t*-test and one-way ANOVA. Results were considered significant when *p* < 0.05.

## Results

### Relationship Between Trypsin Activity and Trypsin Modulating Oostatic Factor Synthesis After the Blood Meal

To find out the relationship between trypsin activity in the gut of female *C. quinquefasciatus* and TMOF circulating in the hemolymph after the blood meal, the guts and hemolymph were assayed for trypsin activity and TMOF concentrations, respectively. Late trypsin activity was first detected at 8 h after the blood meal the activity increased reaching a peak at 30 h and then rapidly declined after 40 h and disappeared at 58 h (Figure 1A). On the other hand, TMOF was first detected in the hemolymph at 30 h reaching a peak between 50 and 60 h and then rapidly declined, reaching a minimum of 100 h after the blood meal (Figure 1B). Since TMOF binds an ABC TMOF gut receptor and it is imported into the gut epithelial cells, the site of trypsin biosynthesis (Borovsky et al., 2021), these results showed that the decline in trypsin activity in the gut of blood-fed female *C. quinquefasciatus* was correlated with the increase of TMOF concentration in the hemolymph.

### Purification of *Culex quinquefasciatus* Late Trypsin

#### Diethylaminoethyl Chromatography

Supernatant that was obtained after homogenizing guts from females that were blood-fed for 30 h was chromatographed on DEAE biogel. Protein absorbance was followed at 280 nm and enzyme activity was followed at 410 nm using BApNA (Borovsky and Schlein, 1988). The column was eluted using a linear gradient of NaCl and fractions (7 ml) were collected. A first peak eluted between 0.05 and 0.150 M NaCl was devoid of trypsin activity. A second peak with low trypsin activity was eluted at 0.22 M NaCl. A third peak eluted between 0.25 and 0.35 M NaCl contained the majority of the trypsin activity. Fractions 60–70 (70 ml) were collected and dialyzed against 50 mM Tris–HCl pH 7.9 at 4°C (Figure 2A).

#### Affinity Chromatography

Affinity chromatography on soybean trypsin inhibitor covalently bound to sepharose has separated the trypsin into two peaks. Specifically, a large protein peak with trypsin activity did not bind to the column, while a small protein peak was retained by the column with high trypsin activity (Figure 2B). The latter protein peak exhibiting trypsin activity and high affinity was collected (12 ml) and the volume was reduced to 1.5 ml using Diaflo concentrating cell.

#### C_18_ High Performance Liquid Chromatography

Aliquots at 200 μl from the concentrated peak after affinity chromatography were purified by HPLC chromatography using reverse phase C_18_ matrix. The column was calibrated with [1,3^3^H]DIP trypsin to identify where trypsin elutes. After HPLC, a single protein peak was identified that corresponded with the [1,3^3^H]DIP-trypsin standard (Figure 2C). After drying the collected trypsin peak under a fine N_2_ stream in a hood, the total protein under the peak was determined to be 200 μg. The HPLC analysis was repeated four more times and the trypsin peaks combined to a final amount of 1 mg. The material was analyzed by automated Edman sequencing yielded an unambiguous N-terminal sequence of the first 19 amino acids of the late *C. quinquefasciatus* trypsin IVGGFEIDILEVPYQISLQ.

### pH Activity Profile

**FIGURE 3:**
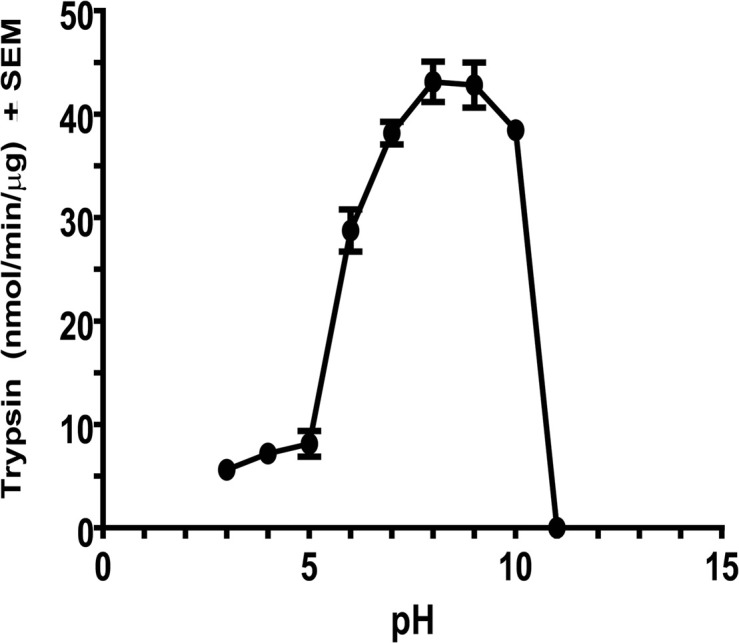
Effect of pH on purified *C. quinquefasciatus* late trypsin activity. Fraction (100 μl) from the combined trypsin peak after affinity chromatography were assayed for trypsin activity using BApNA using 0.2 M citric/phosphate (pH 3.2–7.2), 0.2 M Tris–HCl (pH 7.2–8.9), and 0.2 glycine/NaOH (pH 8.9–10.9). Each assay was repeated three times and is expressed as means ±SEM.

Maximum activity of *C. quinquefasciatus* trypsin after affinity chromatography at different pH values occurred between pH 8 and 9 (Figure 3). Low activity was observed between pH 3 and 5 and about 88% of the maximal activity was observed at pH 7 and 10, whereas no activity was observed at pH 11. These results indicated that the enzyme favored a gut alkaline environment similar to trypsin-like enzymes from *Helicoverpa armigera*, *Heliothis virescens*, and *D. abbreviatus* (Johnston et al., 1991, 1995; Yan et al., 1999).

### Cloning and Sequencing of *Culex quinquefasciatus* Late Trypsin cDNA

**FIGURE 4:**
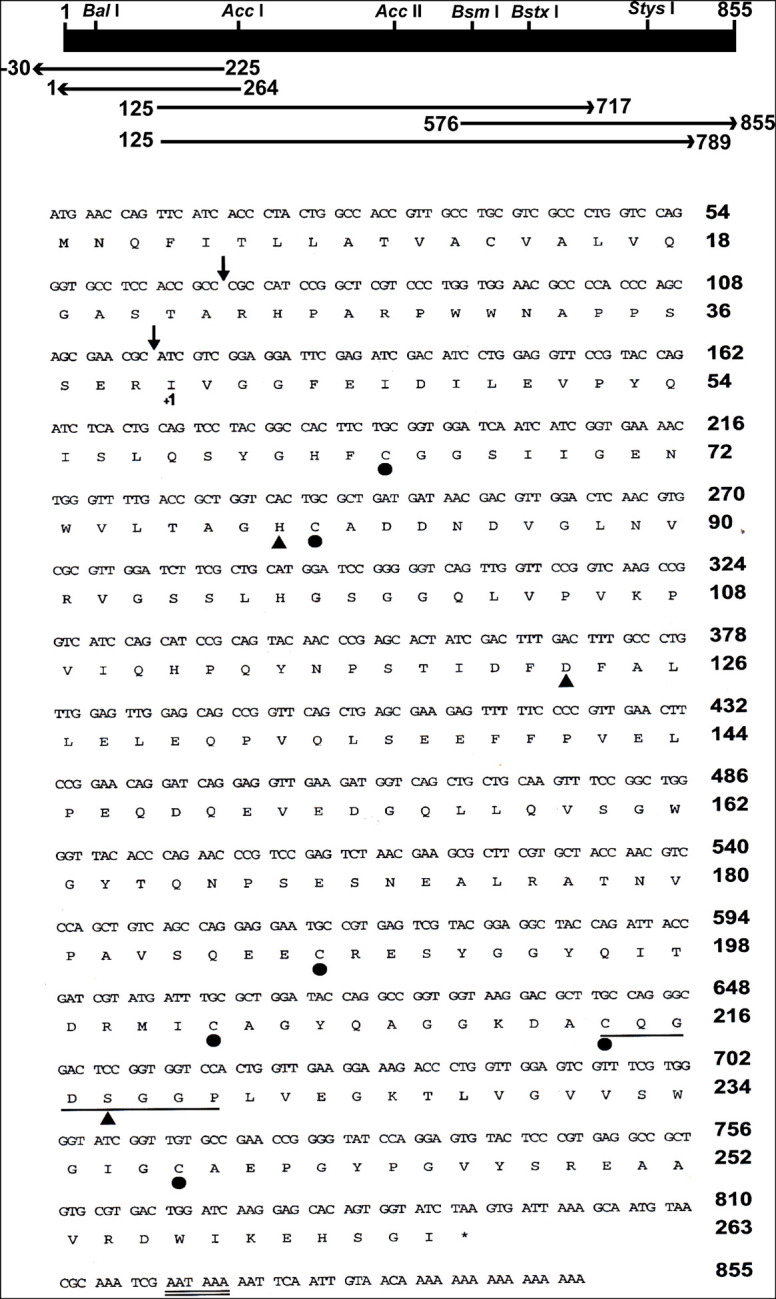
Partial restriction map, cloning and sequencing strategy, and nucleotides sequence, and predicted amino acid sequence of *C. quinquefasciatus* late trypsin. The horizontal bar (top) indicates the restriction enzymes position of the 855 bp late trypsin. Horizontal arrows show the PCR fragments that were amplified, cloned, and sequenced. Predicted sites of cleavage of the signal peptide and activation peptide are denoted by arrows. The conserved sequence around S218 and the polyadenylation signal are underlined by single and double lines, respectively. The position of the catalytic triad residues is denoted by a close triangle and the positions of the six cysteines are marked by close circles. The numbering of the mature enzyme starts at I40 (+1) after the cleavage site of the activation peptide.

**FIGURE 5:**
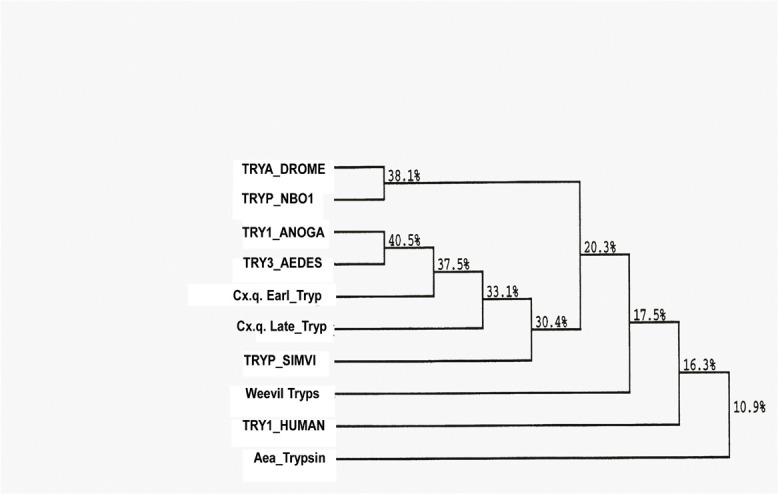
Phylogenetic tree based on multiple alignments according to the Higgins–Sharp algorithm (Clustral 4) of DNAStar v.12. The branching order similarities (%) are based on sequences found at the GenBank.

Five fragments of cDNA were synthesized from late trypsin mRNA extracted from the epithelial cells lining the midguts of female *C. quinquefasciatus* 30 h after the blood meal. Two cDNA fragments of 592 and 664 bp, respectively, of the late trypsin (Figure 4) were reverse transcribed using downstream primers DB 66 and DB 95 and amplified by PCR with upstream primer DB 46 (Table 1). Two cDNA fragments at the 5′ end 264 and 255 bp (Figure 4) were amplified by 5′ RACE (Frohman, 1993) using forward primer dT_17_ adapter and reverse primers DB 80 and DB 73 (Table 1). A cDNA fragment at the 3′ end (279 bp, Figure 4) was amplified by RT-PCR with forward primer DB 75 and reverse primer pair dT_17_ adapter/adapter (Table 1). The five amplified cDNA fragments (Figure 4) were separated by electrophoresis on agarose gel (2%), stained with ethidium bromide, eluted from the agarose using spin columns (Sigma, St. Louis, MO, United States), and further purified on QIAquick columns (Qiagen, Germantown, MD, United States). The five cDNA fragments were subcloned and sequenced. The nucleotide sequence and the deduced amino acid sequence are shown in Figure 4. The 855 bp cDNA nucleotides encoding 263 amino acids open reading frame (ORF) with a methionine codon at position 1 were deposited at the GenBank (accession number U65412). The N-terminal is hydrophobic and represented a signal peptide with a cleaving site after A23 (von Heijne, 1986) that upon cleavage released a trypsinogen with a 16-amino acid activation peptide. Cleavage at R39 released the active late trypsin (Figure 4). The mature enzyme residues I40 to I263 had 224 amino acids and 6 cysteines that could form 3 cysteine bridges. A consensus polyadenylation signal (AATAAA) was found at position 820. The specificity pocket sequence was KDAC (amino acids K211–C214) and the N-terminal IVGG (Figure 4) was similar to the early *C. quinquefasciatus* trypsin (Borovsky et al., 2018). The catalytic active center contained H79, D123, and S218 and was highly conserved in insect serine proteases (Yan et al., 1999). A phylogenetic tree, based on the homology of amino acid sequences of 10 insects (Figure 5) indicated that *C. quinquefasciatus* late trypsin was 33% similar with trypsins of *A. aegypti* and *A. gambiae*, 30% similar with *Simulium*, 20% similar with *Drosophila* and *Neobellieria*, and 16% similar with human trypsin (Figure 5).

### Three-Dimensional Model Analysis

**FIGURE 6:**
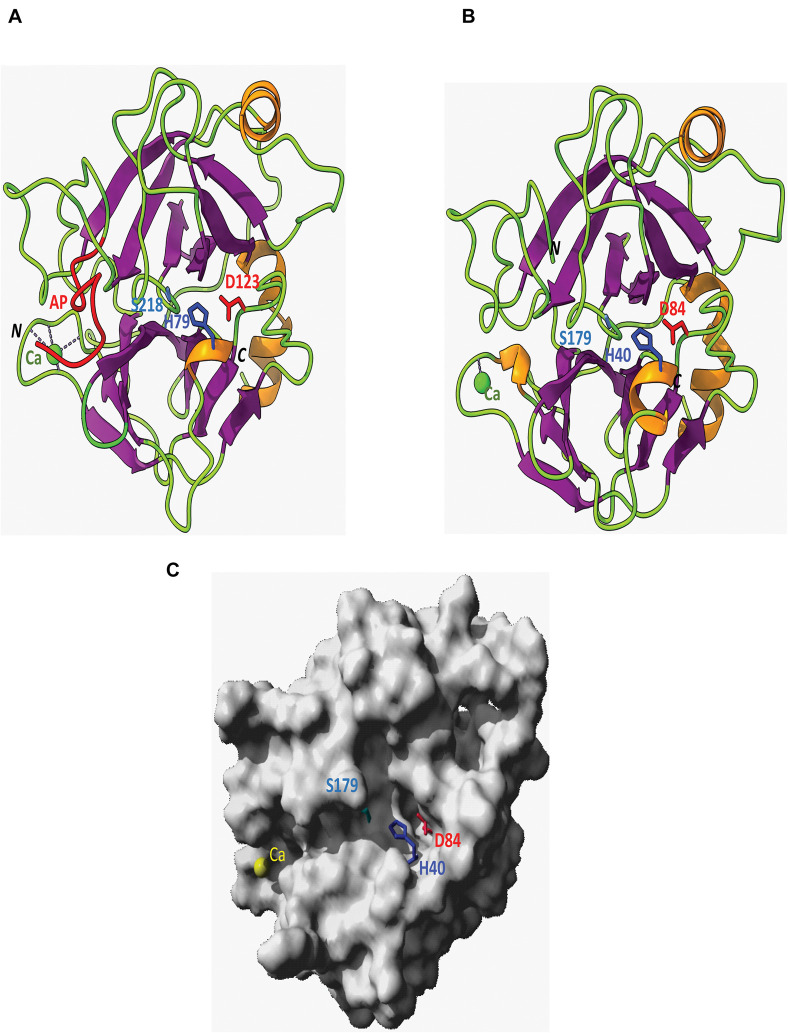
Ribbon-drawn diagrams showing the organization *C. quinquefasciatus* late trypsin. **(A)** Pro-trypsin in two domains delineating a central groove that contains the catalytic triad H79, D123, and S218 of the enzyme. The C-terminal part of the activation peptide (AP) is colored red and the coordinated Ca^2+^ is colored green. **(B)** Ribbon diagram showing the organization of the mature trypsin with the catalytic triad H40, D84, and S179 of the enzyme. The N (N) and C (C) terminals of the pro-trypsin and mature trypsin are shown. **(C)** Molecular surface of the mature trypsin showing the central groove containing the enzyme catalytic triad H40, D84, and S179. The model is similarly oriented as in **(B)** and the coordinated Ca^2+^ is colored yellow.

The ribbon diagrams showed the catalytic triad of H79, D123, and S218 of the pro-trypsin and H40, D84, and S179 for the activated trypsin (Figures 6A,B). The 10 amino acids around S218 in the pro-trypsin and S179 in the active trypsin models were conserved in most of the sequences examined and formed a coil as part of the active site (Figures 6A,B) and sat at the top of the oxygenation hole that was located below similar to other published models of *Neobellieria*, *Drosophila*, and *Diaprepes* trypsins (Borovsky et al., 1996; Yan et al., 1999). A single helix was found at the C terminus of both ribbon diagrams. The active site was situated in a groove between two antiparallel-β-barrel type domains. Domain 1 contained two residues of the catalytic triad, namely H79 and D123 in the pro-trypsin and H40 and D 84 in the active trypsin, whereas S218 in the pro-trypsin and S179 in the active trypsin were in the second domain (Figures 6A,B). These features were also similar in the *Neobellieria* and *Drosophila* models (Borovsky et al., 1996; Yan et al., 1999) and had been reported in models of other trypsins and chymotrypsins which were based on X-ray-diffraction data (Blow, 1976; Branden and Tooze, 1991). The molecular surface of *C. quinquefasciatus* activated trypsin showed a central groove containing the catalytic triad H40, D84, and S179 of the enzyme, illustrating the separation of H40 and D84 from S179 into two domains as mentioned above (Figure 6C).

### Comparison Between the Levels of Trypsin and Its Transcript After the Blood Meal

**FIGURE 7:**
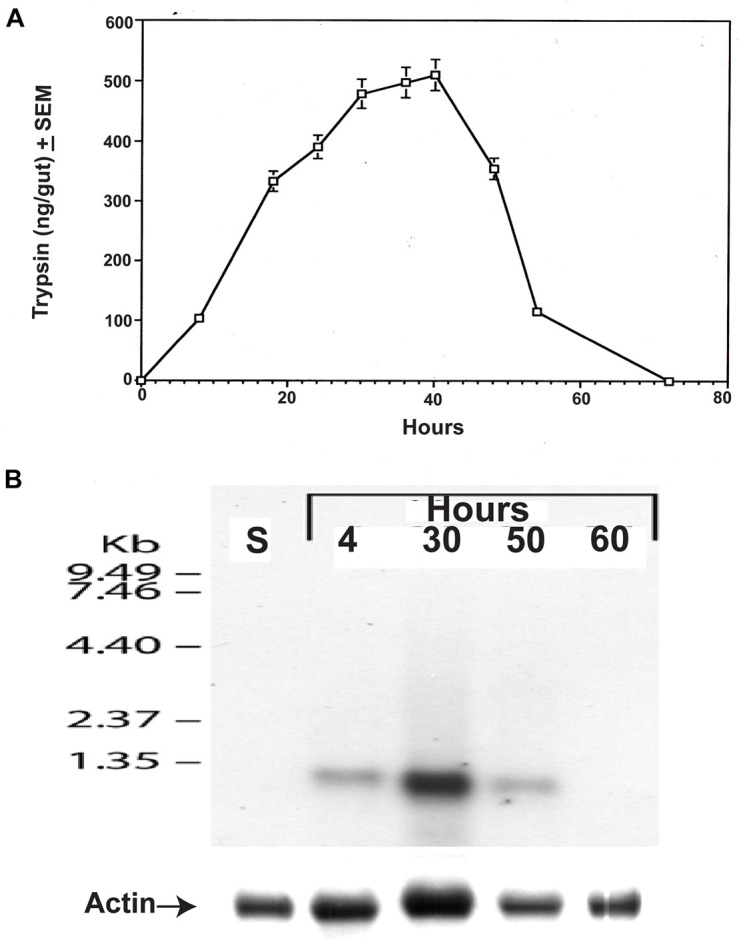
*Culex quinquefasciatus* late trypsin amounts and trypsin transcript in the gut at different times after the blood meal. **(A)** Trypsin amounts (ng/gut) were followed in the gut after the blood meal (Borovsky and Schlein, 1988). The results are expressed as means of three determinations ±SEM. **(B)** Northern blot analysis of the late trypsin transcript in the gut epithelial cells at different times after the blood meal and in sugar-fed female gut epithelial cells (S). Actin was run as a control to show equal transfer and the analysis was repeated two times showing similar results.

Female *C. quinquefasciatus* were fed a blood meal and at intervals, guts were assayed for the amount of the late trypsin in the mentioned body part (Borovsky and Schlein, 1988). At 8 h after the blood meal, the female gut contained 100 ng of trypsin. The amount of trypsin in the gut at 24 h was 325 ng reaching a maximum between 32 and 42 h of 480–510 ng and then declined rapidly to 110 ng at 54 h and disappeared at 72 h (Figure 7A). Northern blot analysis was carried on gut epithelial cells at different times after the blood meal (4, 30, 50, and 60 h) and compared with sugar-fed female gut epithelial cells (control). A single RNA band below 1.35 kb was detected at 4, 30, and 50 h after the blood meal. No trypsin transcript was detected in the guts of sugar-fed females or females that were analyzed at 60 h (Figure 7B). A heavy transcript band was detected at 30 h and lower bands at 4 h and 50 h (Figure 7B), indicating that the amounts of trypsin transcript in the gut epithelial cells and trypsin in the gut were correlated. When the amount of trypsin in the gut rapidly declined at 50 h, the trypsin transcript also decreased and no trypsin transcript was found at 60 h or in sugar-fed controls (Figures 7A,B).

### Effect of Trypsin Modulating Oostatic Factor on Inhibition of Trypsin Translation in the Gut

**FIGURE 8:**
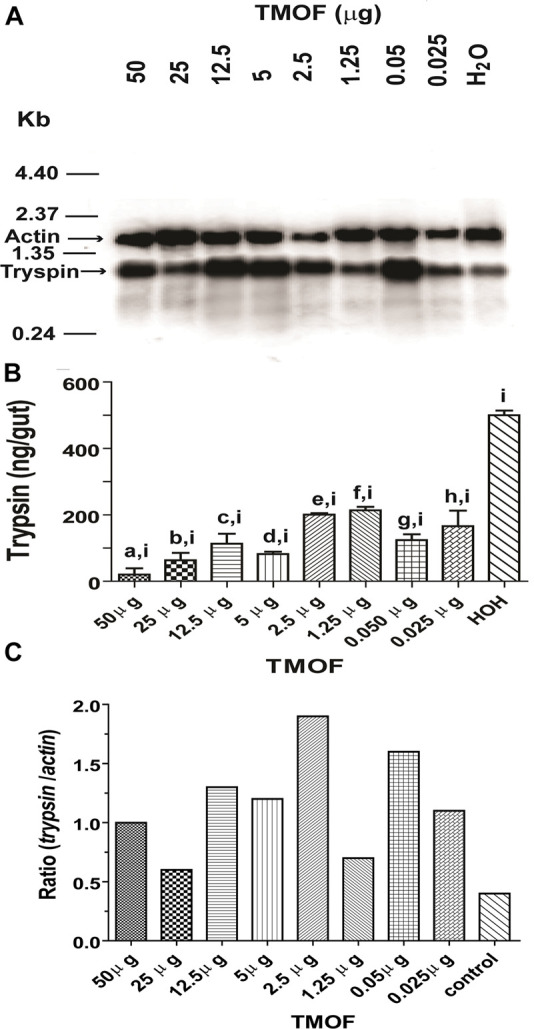
Comparison between *C. quinquefasciatus* late trypsin transcript and trypsin amount in the gut after injection of TMOF to blood-fed females and analyzing the guts at 30 h. **(A)** Northern blot analysis of the late trypsin transcript in gut epithelial cells after injecting different amounts of TMOF (0.025 to 50 μg). Water injection was used as a control and actin to show transfer in all the lanes. The Northern blot was repeated twice showing similar results. **(B)** Trypsin amounts (ng/gut) of gut epithelial cells that were injected with different amounts of TMOF or water control as in **(A)**. Results are expressed as means of 3 determinations ±SEM. Significant differences between different amounts of injected TMOF and water control (*p* < 0.05) were denoted by a combination of two small letters (a,i; b,i; c,i; d,i; e,i; f,i; g,i; h,i). Significant differences in trypsin biosynthesis after injecting different amounts of TMOF into female *C. quinquefasciatus* (a, b, c, d, e, f, g, h, and i) were determined using one-way ANOVA (*p* < 0.0001, *n* = 9, F38.98, *R* = 0.9454). **(C)** The ratios of the *trypsin/actin* transcripts in **(A)** after injecting different amounts of TMOF into *C. quinquefasciatus* were plotted and compared with injections of water control.

Our results showed that the late trypsin activity in the gut was closely correlated with the amount of TMOF circulating in the hemolymph of female *C. quinquefasciatus* after the blood meal (Figure 1). Injections of different amounts of TMOF into female *C. quinquefasciatus* inhibited the translation of the late trypsin transcript in the gut epithelial cells and caused a significant reduction in the amount of trypsin in the gut when compared with controls that were injected with water even though the trypsin transcript was equally synthesized by the gut epithelial cells (Figures 8A,B). Analysis of the results using unpaired two-tailed *t-*tests showed that the injected females synthesized significantly (*p* < 0.05) less trypsin than water-injected controls (Figure 8B). At concentrations that were similar to TMOF concentrations circulating in the hemolymph, injection of TMOF 0.025 μ and 0.05 μg inhibited trypsin in the gut by 72 and 78%, respectively, as compared with the water-injected control group (Figure 8B). One-way ANOVA of the TMOF and water injected groups confirmed that all the means were significantly different from each other (*p* < 0.0001, *n* = 9, F38.98, *R* = 0.9454). These results indicated that TMOF inhibited the translation of the trypsin message in the gut epithelial cells.

## Discussion

The late trypsin of *C. quinquefasciatus* was cloned, characterized and its translational control by TMOF was studied. The late trypsin activity was detected 4 h after the blood meal reaching a peak between 30 and 40 h and disappeared after 58 h (Figure 1A). TMOF is the decapeptide that controls trypsin biosynthesis in mosquitoes and other insects (Borovsky, 1988; Borovsky et al., 1996, 2006). TMOF titer was first detected in the hemolymph at 20 h and increased to a maximum between 50 and 60 h in concert with a rapid decline of trypsin activity in the gut after 44 h (Figure 1). These observations indicated that as TMOF concentration in the hemolymph increases, trypsin activity rapidly declines. A TMOF receptor was located by cytoimmunohistochemistry, cloned, sequenced, and characterized from mosquito gut epithelial cells (Borovsky et al., 1994a, 2021). The TMOF gut receptor is an ABC importer allowing TMOF to enter the gut epithelial cells from the hemolymph and to control trypsin biosynthesis in the epithelial cells. To determine the amino-terminal sequence of the late *C. quinquefasciatus* trypsin cDNA, we purified the enzyme using DEAE chromatography followed with affinity and C_18_ reverse-phase HPLC (Borovsky et al., 1996). N-terminal sequencing after HPLC analysis detected a 19 amino acid sequence (IVGGFEIDILEVPYQISLQ) in which the first 6 amino acids at the N-terminal were similar to the *C. quinquefasciatus* early trypsin (Borovsky et al., 2018), and the first 5 amino acids at the N-terminal sequence were similar with the diapause downregulated trypsin-like enzyme in *C*ule*x pipiens* that showed amino acid similarity (51%) to the *A. aegypti* early trypsin (Robitch and Denlinger, 2005). The late trypsin of *C. quinquefasciatus* is most active at alkaline pH of 8, 9, and 10.4 (Figure 3), indicating that the pH of *C. quinquefasciatus* gut is probably alkaline as was shown for *D. abbreviatus, H. armigera*, and *H. virescens* (Johnston et al., 1991, 1995; Yan et al., 1999).

To study the properties of the *C. quinquefasciatus* late trypsin, a full-length cDNA that encoded the trypsin was sequenced. Degenerate primers (DB 46 and DB 66) from these two regions were designed (Table 1) using the N-terminal sequence that was obtained after three-step column chromatography and sequence similarity around the serine residue in the active site pocket (Figure 4). A cDNA band (592 bp) was identified by agarose gel electrophoresis, purified, sequenced, and used to construct primers to be used for 3′ and 5′ RACE (Frohman, 1993), amplifying the cDNA up to the poly(A) tail (Figure 4). The deduced amino acid sequence that encoded a proenzyme of 263 amino acids (M_*r*_ 28,320) was longer than was reported for trypsins from *N. bullata*, *M. sexta*, and *D. abbreviatus* (Peterson et al., 1994; Borovsky et al., 1996; Yan et al., 1999). It contained a signal peptide of 23 amino acids with a cleavage site between A23 and R24 that liberated a trypsinogen with 16 amino acid activation peptides (Figures 4, 6A). For the early trypsin of *C. Quinquefasciatus*, a 21 amino acid activation peptide was suggested, whereas for *A. aegypti*, *D. melanogaster*, and *N. bullata*, 10-6 amino acid activation peptide was suggested (Barillas-Mury et al., 1991; Borovsky et al., 1996; Noriega et al., 1996). *C. Quinquefasciatus* late trypsin has an even number of cysteines (six), whereas *C. quinquefasciatus* early trypsin has an uneven number of cysteines (seven) similar to the early trypsin of *A. aegypti* (Kalhok et al., 1993; Borovsky et al., 2018). *C. quinquefasciatus* late trypsin has positively charged amino acids (9 arginines and 6 histidines) and negatively charged amino acids (12 aspartic acids, 21 glutamic acids, 7 cysteines, and 9 tyrosines) (Figure 4). The multiple-sequence alignment shows that the amino acid sequence of the *C. quinquefasciatus* late trypsin has 30.4% similarity with the early trypsin and 33% similarity to the trypsin 3 of *A. aegypti* and lower similarities with human trypsin 1 (10.9%) (Figure 5). The three-dimension built model of *C. quinquefasciatus* exhibited the antiparallel-β-barrel characteristic of the chymotrypsin superfamily that included trypsin, chymotrypsin, elastase, and thrombin (Branden and Tooze, 1991) and was shown in three-dimension models for *C. quinquefasciatus* early trypsin and *Neobellieria* trypsin (Yan et al., 1999; Borovsky et al., 2018).

The late trypsin of *C. quinquefasciatus* was stimulated by the blood meal, wherein 8 h after the blood meal, 100 ng of the late trypsin was found in the gut of female *C. quinquefasciatus*. The Northern blot analysis corroborated this result showing a faint trypsin band of the late trypsin even earlier at 4 h (Figures 7A,B). Our Northern probe (294 bp amplicon; Table 1) was made specific by starting 30 nucleotides before the ATG signal (Supplementary Material) and, thus, was not similar to the *C. quinquefasciatus* early trypsin sequence (accession number AY029276; Borovsky et al., 2018). Indeed, Northern blot analysis of gut epithelial cells of sugar-fed females did not show a band after probing the blot with the radioactively labeled probe (Figure 7B). A faint band was observed at 50 h and no band at 60 h. Our actin probe showed that the transfer was equal throughout the lanes suggesting that the late trypsin is not synthesized at that time in agreement with our earlier results showing no enzyme activity in the gut and maximal TMOF concentration in the hemolymph (Figure 1). These observations suggested that TMOF modulates trypsin biosynthesis in the gut as was shown in mosquitoes and *Neobellieria* (Borovsky et al., 1990, 1993, 1994b, 1996; Bylemans et al., 1994; De Loof et al., 1995). Injection of different concentrations of TMOF (0.025–50 μg) prevented the translation of the trypsin transcript and the synthesis of trypsin by the mosquito gut at 30 h as compared with controls that were injected with water (Figure 8). TMOF did not cause a reduction of the trypsin transcript by stimulating, for example, nucleases (Schoenberg and Maquat, 2012; Figure 8A). The level of the trypsin transcript, after physiological concentrations of TMOF (0.025 and 0.05 μg) were injected, was much higher than the trypsin transcript of the control. To compare between the *trypsin* and *actin* transcripts after the injections of different amounts of TMOF the *trypsin* and *actin* transcripts intensities on the Northern blot were scanned and the ratios were calculated to eliminate uneven transfer of the actin bands. Indeed, in all cases, the *trypsin/actin* ratio is several-fold higher than the control, and after injecting physiological amounts of TMOF (0.025 and 0.05 μg) the differences are 2.8- and 4-fold, respectively higher as compared with the water injected control (Figure 8C). These results indicated that after injecting TMOF to blood-fed female *C. quinquefasciatus* and although the gut epithelial cells synthesized higher amounts of the trypsin transcript, the amount of trypsin that was translated by the epithelial cells after injecting TMOF (0.025 and 0.05 μg) was three- and fourfold lower, respectively, than after water injection (control) (Figure 8B). Similar results were found when 50 μg TMOF a 2,000-fold higher amount that is circulating in the hemolymph was injected (Figures 1A, 8B,C). TMOF did not cause a decrease in the trypsin transcript abundance in the midgut epithelial cells by inducing exonucleases that caused mRNA decay (Schoenberg and Maquat, 2012). We searched the *C. quinquefasciatus* genome at NCBI^1^ to find out if all the enzymes that regulate mRNA decay could be located in the *C. quinquefasciatus* transcriptome. Indeed, PARN the exonuclease that shortened the poly(A) tail by the deadenylase poly(A) ribonuclease (LOC6051824) was located on chromosome 3. Decapping enzyme that hydrolyzes the cap (LOC6045051) was found on chromosome 2. The enzymes that degraded the body of the mRNA from the 5′ to 3′ XRN1 (LOC 6039697 and LOC 6047830) was located on chromosomes 3 and 1, respectively, including the exosome complex exonuclease RPP44 (LOC6035404) which was located on chromosome 1 that degraded the mRNA from 3′ to 5′. Since the whole exonuclease decay pathway was found in *C. quinquefasciatus* genome, and since our Northern blot analysis (Figure 8A) indicated that the message has not been hydrolyzed by the exonuclease decay pathway, it was highly likely that TMOF affected directly or indirectly the translation of trypsin in the gut epithelial cells. Translational control had been reported for ferritin, transferrin receptor, ribonucleotide reductase, heat shock proteins, tubulin, and oocyte protein, to name a few (Lindquist, 1986; Berry et al., 1988; Yen et al., 1988; Theil, 1990; Sachs and Wahle, 1993). More work is currently in progress in our laboratory to find out how TMOF modulates the translation of *C. quinquefasciatus* transcript in the gut epithelial cells.

## Data Availability Statement

The datasets presented in this study can be found in online repositories. The names of the repository/repositories and accession number(s) can be found below: https://www.ncbi.nlm.nih.gov/genbank/; Accession Number: U65412.

## Author Contributions

DB did the research and wrote the manuscript. PV did the research and analyzed the results and wrote the manuscript. PR prepared the three-dimensional models and analyzed the results. CP did the research and analyzed the results. AD analyzed the results and wrote the manuscript. All authors contributed to the article and approved the submitted version.

## Conflict of Interest

PV was employed by the company ProteoFormiX BV. The remaining authors declare that the research was conducted in the absence of any commercial or financial relationships that could be construed as a potential conflict of interest.

## Publisher’s Note

All claims expressed in this article are solely those of the authors and do not necessarily represent those of their affiliated organizations, or those of the publisher, the editors and the reviewers. Any product that may be evaluated in this article, or claim that may be made by its manufacturer, is not guaranteed or endorsed by the publisher.

## Footnotes

1
https://www.ncbi.nlm.nih.gov/search/all/?term=Culex%20quinquefasciatus%20

## References

[B1] Barillas-Mury C. V. Noriega F. G. Wells M. A. (1995). Early trypsin activity is part of the signal transduction system that activates transcription of the late trypsin gene in the midgut of the mosquito Aedes aegypti. *Insect Biochem. Mol. Biol.* 25 241–246. 10.1016/0965-1748(94)00061-l 7711754

[B2] Barillas-Mury C. Graf R. Hagedorn H. H. Wells M. A. (1991). cDNA and deduced amino acid sequence of a blood meal-induced trypsin from the mosquito, Aedes aegypti. *Insect Biochem.* 21 825–831. 10.1016/0020-1790(91)90089-w

[B3] Benkert P. Biasini M. Schwede T. (2011). Toward the estimation of the absolute quality of individual protein structure models. *Bioinformatics* 27 343–350. 10.1093/bioinformatics/btq662 21134891 PMC3031035

[B4] Berry J. O. Carr J. P. Klessig D. F. (1988). mRNAs encoding ribulose-1,5-bisphosphate carboxylase remain bound to polysomes but are not translated in amaranth seedlings transferred to darkness. *Proc. Natl Acad. Sci. U S A.* 85 4190–4194. 10.1073/pnas.85.12.4190 16593940 PMC280392

[B5] Blow D. M. (1976). Structure and mechanism of chymotrypsin. *Acc. Chem. Res.* 9 145–152. 10.1021/ar50100a004

[B6] Borges-Veloso A. Saboia-Vahia L. Dias-Lopes G. Domont G. B. Britto C. Cuervo P. (2015). In-depth characterization of trypsin-like serine peptidases in the midgut of the sugar fed Culex quinquefasciatus. *Parasites Vectors* 8:373. 10.1186/s13071-015-0985-0) 26174750 PMC4502911

[B7] Borovsky D. (1988). Oostatic hormone inhibits biosynthesis of midgut proteolytic enzymes and egg development in mosquitoes. *Arch. Insect Biochem. Physiol.* 7 187–210. 10.1002/arch.940070305

[B8] Borovsky D. (2003). Biosynthesis and control of mosquito gut proteases. *IUBMB Life* 55 435–441. 10.1080/15216540310001597721 14609198

[B9] Borovsky D. Mahmood F. (1995). Feeding the mosquito *Aedes aegypti* with TMOF and its analogs, effect on trypsin biosynthesis and egg development. *Reg. Peptides* 57 273–281. 10.1016/0167-0115(95)00041-9 7480877

[B10] Borovsky D. Schlein Y. (1988). Quantitative determination of trypsinlike and chymotrypsinlike enzymes in insects. *Arch. Insect Biochem. Physiol.* 8 249–260. 10.1002/arch.940080406

[B11] Borovsky D. Carlson D. A. Griffin P. R. Shabanowitz J. Hunt D. F. (1990). Mosquito oostatic factor a novel decapeptide modulating trypsin-like enzyme biosynthesis in the midgut. *FASEB J.* 4 3015–3020. 10.1096/fasebj.4.12.2394318 2394318

[B12] Borovsky D. Carlson D. A. Griffin P. R. Shabanowitz J. Hunt D. F. (1993). Mass spectrometry and characterization of *Aedes aegypti* trypsin modulating oostatic factor (TMOF) and its analogs. *Insect Biochem. Mol. Biol.* 23 703–712. 10.1016/0965-1748(93)90044-s 8353526

[B13] Borovsky D. Deckers K. Vanhove A. C. Verstraete M. Rouge P. Shatters R. G. Jr. (2021). Cloning and Characterization of *Aedes aegypti* Trypsin Modulating Oostatic Factor (TMOF) Gut-Receptor. *Biomolecules* 11:934. 10.3390/biom11070934 34201823 PMC8301768

[B14] Borovsky D. Hancock R. G. Rouge P. Powell C. A. Shatters R. G. Jr. (2018). Juvenile hormone affects the splicing of *Culex quinquefasciatus* early trypsin messenger RNA. *Arch. Insect Biochem. Physiol.* 2018:e21506. 10.1002/arch.21506 30176073

[B15] Borovsky D. Janssen I. Vanden Broeck J. Huybrechts R. Verhaert P. DeBondt H. L. (1996). Molecular sequencing and modeling of *Neobellieria bullata* trypsin: Evidence for translational control with Neb TMOF. *Eur. J. Biochem.* 237 279–287. 10.1111/j.1432-1033.1996.0279n.x 8620885

[B16] Borovsky D. Powell C. A. Carlson D. A. (1992). Development of specific RIA and ELISA to study trypsin modulating oostatic factor in mosquitoes. *Arch. Insect Biochem. Physiol.* 21 13–21. 10.1002/arch.940210103 1421442

[B17] Borovsky D. Powell C. A. Nayar J. K. Blalock J. E. Hayes T. K. (1994a). Characterization and localization of mosquito-gut receptors for trypsin modulating oostatic factor (TMOF) using a complementary peptide and immunocytochemistry. *FASEB J.* 8 350–355. 10.1096/fasebj.8.3.7908271 7908271

[B18] Borovsky D. Rabindran S. Dawson W. O. Powell C. R. Iannotti D. Morris T. (2006). Expression of *Aedes* TMOF on the virion of TMV: potential larvicide. *Proc. Nat. Acad. Sci.* 103 18963–18968. 10.1073/pnas.0606146103 17148608 PMC1748160

[B19] Borovsky D. Song Q. Ma M. Carlson D. A. (1994b). Biosynthesis, secretion and cytoimmunochemistry of trypsin modulating oostatic factor of *Aedes aegyptis*. *Arch. Insect Biochem. Physiol.* 27 27–38.

[B20] Bradford M. (1976). A rapid and sensitive method for the quantification of microgram quantities of protein utilizing the principal protein-dye binding. *Anal. Biochem.* 72 248–254. 10.1006/abio.1976.9999 942051

[B21] Branden C. Tooze J. (1991). *Introduction to Protein Structure.* New York, NY: Garland Publishing, Inc.

[B22] Bylemans D. Borovsky D. Hunt D. F. Shabanowitz J. Grauwels L. De Loof A. (1994). Sequencing and characterization of trypsin modulating oostatic factor (TMOF) from the ovaries of the grey fleshfly. *Neobellieria Bullata Regul. Pept.* 50 61–72. 10.1016/0167-0115(94)90192-9 8159807

[B23] Davis C. A. Riddler D. C. Higgins M. J. Holden J. J. A. White B. N. (1985). A gene family in *Drosophila melanogaster* coding for trypsin-like enzymes. *Nucleic Acids Res.* 13 6605–6619. 10.1093/nar/13.18.6605 2414727 PMC321980

[B24] De Loof A. Bylemans D. Schoofs L. Janssen I. Spittaels K. Vanden Broeck J. (1995). Folliculostatins, gonadotropins and a model for control of growth in the grey fleshfly *Neobellieria (Sarcophaga) bullata, Insect Biochem*. *Molec. Biol.* 25 661–667. 10.1016/0965-1748(95)00005-g 7627197

[B25] Farajollahi A. Fonseca D. M. Kramer L. D. Kilpatrick A. M. (2011). Bird biting mosquitoes and human disease: a review of the role of *Culex Pipiens* complex mosquitoes in epidemiology. *Infect. Genet. Evol.* 11 1577–1585. 10.1016/j.meegid.2011.08.013 21875691 PMC3190018

[B26] Felix C. R. Betschart B. Billingsley P. F. Freyvogel T. A. (1991). Post−feeding induction of trypsin in the midguts of *Aedes aegypti* L. (Diptera: Culicidae) is separable into two cellular phases. *Insect Biochem.* 21 197–203. 10.1016/0020-1790(91)90050-o

[B27] Frohman M. A. (1993). Rapid amplification of complementary DNA ends for generation of full-length complementary DNAs: thermal RACE. *Methods Enzymol.* 8 340–356. 10.1016/0076-6879(93)18026-9 7685466

[B28] Grosdidier A. Zoete V. Michielin O. (2011). SwissDock, a protein-small molecule docking web service based on EADock DSS. *Nucleic Acids Res.* 39 W270–W277. 10.1093/nar/gkr366 21624888 PMC3125772

[B29] Johnston K. A. Lee M. J. Brough C. Hilder V. A. Gatehouse A. M. R. Gatehouse J. A. (1995). Protease activities in the larval midgut of *Heliothis virescens*: Evidence for trypsin and chymotrypsin like enzyme. *Insect Biochem. Molec. Biol.* 25 375–383. 10.1016/0965-1748(94)00077-u

[B30] Johnston K. A. Lee M. J. Gatehouse J. A. Anstee J. H. (1991). The partial purification and characterization of serine protease activity in midgut of larval *Helicoverpa armigera*. *Insect Biochem.* 21 389–397. 10.1016/0020-1790(91)90005-y

[B31] Kalhok S. E. Tabak L. M. Prosser D. E. Brook W. Downe A. E. R. White B. N. (1993). Isolation, sequencing and characterization of two cDNA clones coding for-trypsin-like enzymes from the midgut of Aedes aegypti. *Insect Mol. Biol.* 2 71–79. 10.1111/j.1365-2583.1993.tb00127.x 9087545

[B32] Krieger E. Koraimann G. Vriend G. (2002). Increasing the precision of comparative models with YASARA NOVA - a self-parameterizing force field. *Proteins* 47 393–402. 10.1002/prot.10104 11948792

[B33] Laskowski R. A. MacArthur M. W. Moss D. S. Thornton J. M. (1993). PROCHECK: a program to check the stereochemistry of protein structures. *J. Appl. Cryst.* 26 283–291. 10.1107/s0021889892009944

[B34] Leiros H. K. McSweeney S. M. Smalås A. O. (2001). Atomic resolution structures of trypsin provide insight into structural radiation damage. *Acta Crystallogr. D Biol. Crystallogr.* 57 488–497. 10.1107/s0907444901000646 11264577

[B35] Leiros H.-K. S. Brandsdal B. O. Andersen O. A. Os V. Leiros I. Helland R. (2004). Trypsin specificity as elucidated by LIE calculations, X-ray structures, and association constant measurements. *Protein Sci.* 13 1056–1070. 10.1110/ps.03498604 15044735 PMC2280040

[B36] Lindquist S. (1986). The heat shock response. *Annu. Rev. Biochem.* 55 1151–1191. 2427013 10.1146/annurev.bi.55.070186.005443

[B37] Lu S. Pennington J. Stonehouse A. Mobula M. Wells M. (2006). Reevaluation of the role of early trypsin activity in the transcriptional activation of the late trypsin gene in the mosquito *Aedes aegypti*. *Insect Biochem. Molec. Biol.* 36 336–343. 10.1016/j.ibmb.2006.01.011 16551547

[B38] Melo F. Feytmans E. (1998). Assessing protein structures with a non-local atomic interaction energy. *J. Mol. Biol.* 277 1141–1152. 10.1006/jmbi.1998.1665 9571028

[B39] Muller H.-M. Crampton J. M. della Torre A. Sinden R. Crisanti A. (1993). Member of a trypsin gene family in *Anopheles gambiae* are induced in the gut by blood meal. *EMBO J.* 12 2891–2900. 10.1002/j.1460-2075.1993.tb05951.x 8335004 PMC413542

[B40] Noriega F. G. Wells M. A. (1999). A molecular view of trypsin synthesis in the midgut of Aedes aegypti. *J. Insect Physiol.* 45 613–620. 10.1016/s0022-1910(99)00052-9 12770346

[B41] Noriega F. G. Pennington J. E. Barillas-Mury C. Wang X. Y. Wells M. A. (1996). Aedes aegypti midgut early trypsin is post-transcriptionally regulated by blood feeding. *Insect Mol. Biol.* 5 25–29. 10.1111/j.1365-2583.1996.tb00037.x 8630532

[B42] Peterson A. M. Barillas-Mury C. V. Well M. A. (1994). Sequence of three cDNAs encoding an alkaline midgut trypsin from *Manduca sexta*. *Insect Biochem. Molec. Biol.* 24 463–471. 10.1016/0965-1748(94)90041-8 8205142

[B43] Robitch R. M. Denlinger D. L. (2005). Diapause in the mosquito *Culex pipiens* evokes a metabolic switch from blood feeding to sugar gluttony. *PNAS* 102 15912–15917. 10.1073/pnas0507958102) 16247003 PMC1276097

[B44] Sachs A. Wahle E. (1993). Poly(A) tail metabolism and function in Eucaryotes. *J. Biol. Chem.* 268 22955–22958. 10.1016/s0021-9258(19)49408-8 8226806

[B45] Schoenberg D. Maquat L. E. (2012). Regulation of cytoplasmic mRNA decay. *Nat. Rev. Genet.* 13 246–259. 10.1038/nrg3160 22392217 PMC3351101

[B46] Simpson J. E. Hurtado P. J. Medlock J. Molaei G. Andreadis T. G. Galvani A. P. (2012). Vector host-feeding preferences drive transmission of multi-host pathogens: West Nile virus as a model system. *Proc. Biol. Sci.* 279 925–933. 10.1098/rspb.2011.1282 21849315 PMC3259921

[B47] Theil E. C. (1990). Regulation of ferritin and transferrin receptor mRNAs. *J. Biol. Chem.* 265 4771–4774. 10.1016/s0021-9258(19)34036-0 2156853

[B48] Toyota E. Iyaguchi D. Sekizaki H. Tateyama M. Ng K. K. S. (2009). A structural comparison of three isoforms of anionic trypsin from chum salmon (*Oncorhynchus keta*). *Acta Crystallogr. D Biol. Crystallogr.* 65 717–723. 10.1107/S0907444909012165 19564692

[B49] von Heijne G. (1986). New method for predicting signal sequence signal sequence cleavage sites. *Nucleic Acids Res.* 14 4683–4690. 10.1093/nar/14.11.4683 3714490 PMC311474

[B50] Yan X.-H. DeBond H. L. Powell C. A. Bullock R. C. Borovsky D. (1999). Sequencing and characterization of the citrus weevil *Diaprepes abbreviatus*, trypsin cDNA; effect of *Aedes* trypsin modulating oostatic factor on trypsin biosynthesis. *Eur. J. Biochem.* 262 627–636. 10.1046/j.1432-1327.1999.00411.x 10411621

[B51] Yen T. J. Machlin P. S. Cleveland D. W. (1988). Autoregulated instability of p-tubulin mRNAs by recognition of the nascent amino terminus of /3-tubulin. *Nature* 334 580–585. 10.1038/334580a0 3405308

