# Supplementary figures and images for: Culex quinquefasciatus Late Trypsin Biosynthesis Is Translationally Regulated by Trypsin Modulating Oostatic Factor
Source: Front Physiol. 2021 Nov 17;12:764061. doi: 10.3389/fphys.2021.764061 (PMC8637831; doi:10.3389/fphys.2021.764061)

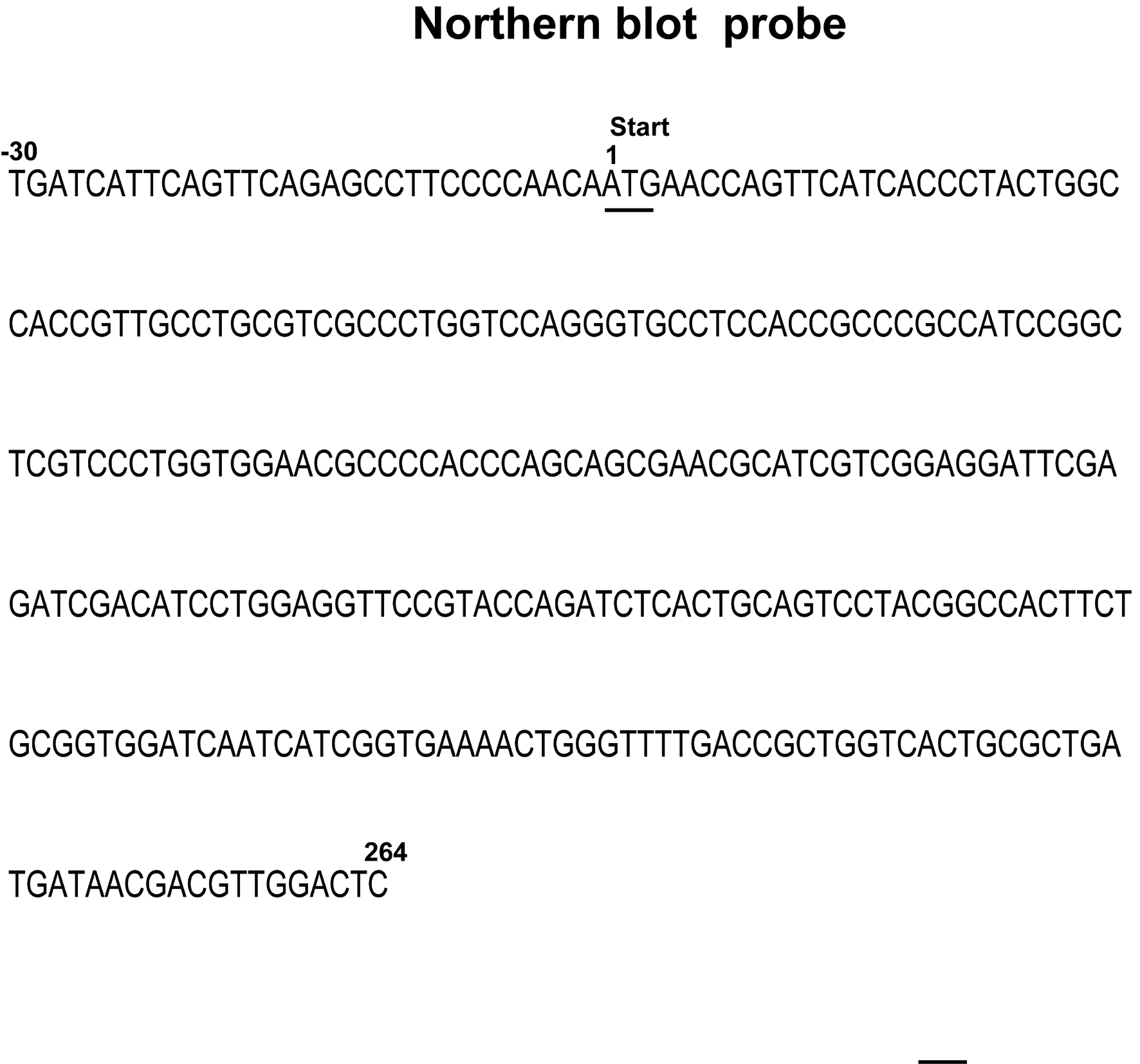

Supplement: Supplementary file 1 [file Image_1.TIF]

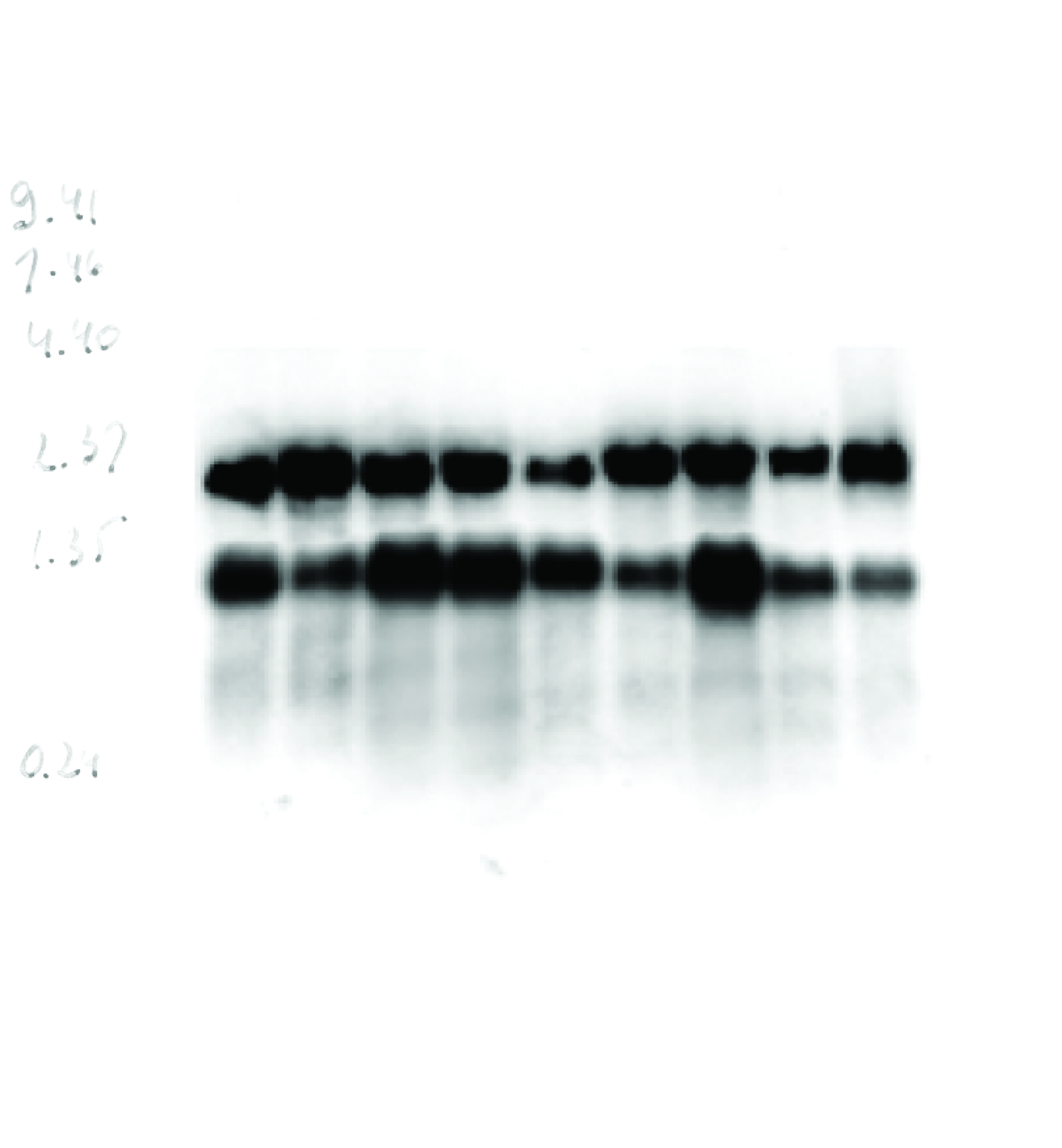

Supplement: Supplementary file 2 [file Image_2.TIF]

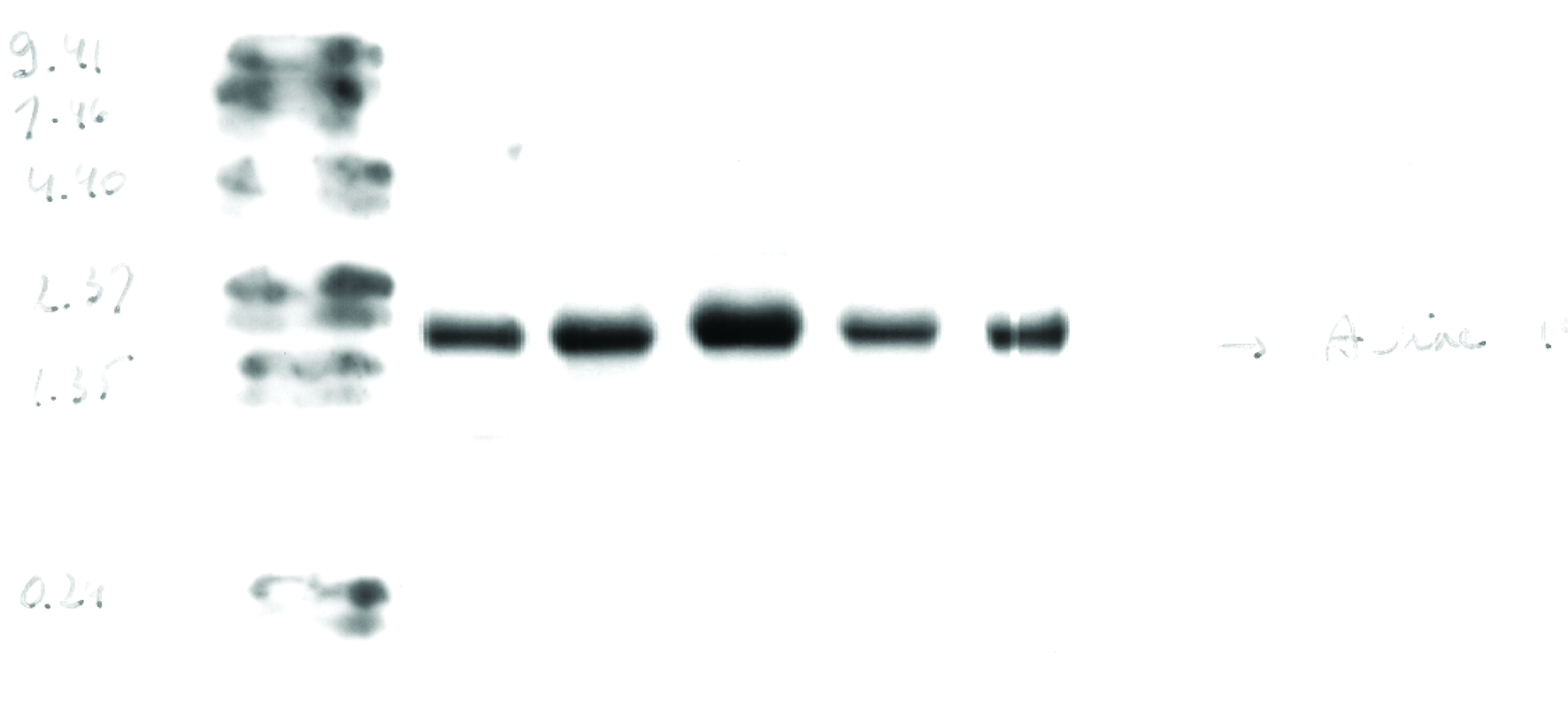

Supplement: Supplementary file 3 [file Image_3.TIF]
